# Recent Advances in Nanoparticle Concentration and Their Application in Viral Detection Using Integrated Sensors

**DOI:** 10.3390/s17102316

**Published:** 2017-10-11

**Authors:** Brian M. Dincau, Yongkuk Lee, Jong-Hoon Kim, Woon-Hong Yeo

**Affiliations:** 1 School of Engineering and Computer Science, Washington State University, Vancouver, WA 98686, USA; brian.dincau@wsu.edu; 2 George W. Woodruff School of Mechanical Engineering, College of Engineering, Georgia Institute of Technology, Atlanta, GA 30313, USA; yklee@gatech.edu; 3 Bioengineering Program, Petit Institute for Bioengineering and Bioscience, Center for Flexible Electronics, Institute for Engineering and Nanotechnology, Institute for Bioengineering & Bioscience, Neural Engineering Center, Georgia Institute of Technology, Atlanta, GA 30332, USA

**Keywords:** biosensors, nanoparticle concentration, viral detection, sensitivity, selectivity

## Abstract

Early disease diagnostics require rapid, sensitive, and selective detection methods for target analytes. Specifically, early viral detection in a point-of-care setting is critical in preventing epidemics and the spread of disease. However, conventional methods such as enzyme-linked immunosorbent assays or cell cultures are cumbersome and difficult for field use due to the requirements of extensive lab equipment and highly trained personnel, as well as limited sensitivity. Recent advances in nanoparticle concentration have given rise to many novel detection methodologies, which address the shortcomings in modern clinical assays. Here, we review the primary, well-characterized methods for nanoparticle concentration in the context of viral detection via diffusion, centrifugation and microfiltration, electric and magnetic fields, and nano-microfluidics. Details of the concentration mechanisms and examples of related applications provide valuable information to design portable, integrated sensors. This study reviews a wide range of concentration techniques and compares their advantages and disadvantages with respect to viral particle detection. We conclude by highlighting selected concentration methods and devices for next-generation biosensing systems.

## 1. Introduction

Recent advances in nanotechnology have enabled the manipulation of nanoscale particles, ranging from synthesized materials including nanoparticles, nanotubes, and quantum dots, to bioparticles such as DNA, proteins, and viruses [1]. Nanomaterials and nanostructures have been widely used to design new biosensors and bioelectronics due to their ability to enhance sensitivity and the potential for developing high-performance sensing systems. The main advantage stems from their high surface area for enhanced interactions with targeted nanoscale particles [2]. Consequently, new methods and systems to detect nanoparticles have gained great attention in disease diagnostics and health monitoring. One important application is to target viral particles in body fluids, including whole viruses, genomic material, and complementary antibodies, via the development of new diagnostic systems.

Infectious diseases caused by viruses (HIV, influenza, and hepatitis) account for nearly 8 million human deaths each year [3]. Early diagnostics are crucial to avoid the spread of viral diseases on a regional level and prevent further harm or even death on an individual level. Accurate and rapid detection of such diseases requires high sensitivity of biosensors due to the relatively low concentration of target viral particles in body fluids, and rapid processing time to ensure timely treatment of the affected individual. Furthermore, the limited resources and required medical personnel in a point-of-care setting can be a significant challenge for the early diagnosis. Thus, simple and inexpensive yet sensitive diagnostic tools are urgently needed to enable timely diagnosis of infectious disease. Many conventional viral assays, however, are unable to satisfy all requirements. The most established method for viral detection is an enzyme-linked immunosorbent assay (ELISA), in which a solid-phase enzyme detects the presence of a particular substance (e.g., antigen). The problem of ELISA is that this method requires specific laboratory equipment and typical sample preparation takes four hours or more, making ELISA impractical for rapid diagnostics [4]. A cell culture or plaque assay, wherein a potentially infected sample is inoculated onto a layer of host cells and observed for unique cytopathic effects [5], is another clinical technique for viral detection and quantification. Even though this method is sensitive, the major drawback is the assay time, often requiring several weeks. In addition, there are several other conventional assays including real-time quantitative reverse transcription polymerase chain reaction (RT-qPCR), hemagglutination, and endpoint dilution. However, all of these heavily rely on diffusion-limited biochemical amplification to indicate the presence of a virus, which requires extensive assay time and larger sample volumes. Thus, these methods are not applicable for on-site, immediate detection of viral particles to prevent epidemics and the spread of disease.

To overcome the aforementioned issues, an alternative way is needed to offer portable, rapid, and sensitive detection of viral particles. Recent studies [2,6] demonstrate novel biosensors, capable of direct, fast, and specific detection of viral targets by using active concentration methodologies. The most important capability to enable the next generation viral assay is the active, controllable manipulation of targets, even within a small sample volume. Here, this review summarizes well-characterized, concentration methods of nanoparticles (NPs) and their applications for viral detection, based on the mechanism via diffusion, centrifugation and microfiltration, electric and magnetic fields, and nano-microfluidic devices. All of these methods focus on concentrating viral particles with the assistance of other synthetic nanoparticles. In addition, while novel concentration techniques have developed for highly sensitive and rapid detection, they are still reliant on cumbersome sample preparation with laboratory equipment, which may not be used in a point-of-care setting. Therefore, we review the state-of-the-art emerging technologies of portable, lab-on-a-chip (LOC) biosensors and bioelectronics, which address the logistical shortcomings of these concentration techniques. 

## 2. Review of Concentration Methods and Relevant Theory 

### 2.1. Diffusion

Diffusion describes the random migration of particles in a solution from high to low concentration zones. In general, diffusion of particles in a medium can be described by Fick’s second law [7]:

(1) $$\frac{\partial c}{\partial t}=D{𝛻}^{2}c,$$

where *c* is the nanoparticle concentration, *t* is time, and *D* is the diffusion coefficient. This equation predicts how diffusion causes the concentration to change with time. 

**Figure 1:**
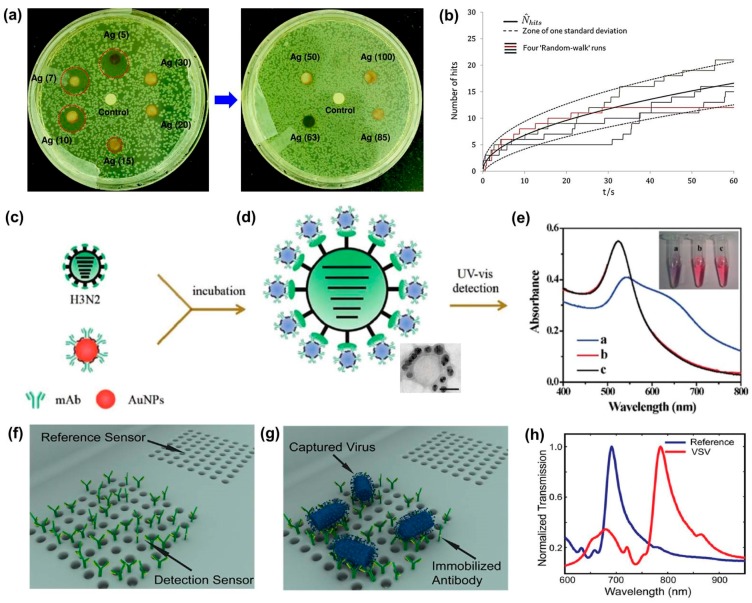
(**a**) photo of a disk diffusion test for a variety of different sized silver nanoparticles against the *E. coli* MTCC 443 strain (reproduced from Agnihotri et al. [8]); (**b**) graph depicting four random-walk simulation runs. The solid black line represents the cumulative number of total hits, while the dashed lines represent zone standard deviation (reproduced from Eloul et al. [11]); (**c**–**e**) flow chart describing the colorimetric detection of influenza virus particles (H3N2) using functionalized gold nanoparticles (AuNPs) (reproduced from Liu et al. [15]); (**c**) the infected sample and functionalized AuNPs are mixed and incubated for 30 min; (**d**) H3N2 and AuNPs bind due to the antibody–antigen interaction, with tunneling electron microscope (TEM) image of resulting aggregate below; (**e**) rearrangement of AuNPs around the viral particles results in a blue shift with intensity that correlates with H3N2 concentration; (**f**–**h**) illustration depicting a nanohole detection sensor and associated spectral response curve: (**f**) detection sensor with antibody; (**g**) capture of vesicular stomatitis virus (VSV) on the sensor; and (**h**) shift of plasmon resonance due to the accumulation of viral particles (reproduced from Yanik et al. [26]).

For example, optical images in Figure 1a [8] show a diffusion test of different sized silver nanoparticles (AgNPs) against an E. coli Microbial Type Culture Collection (MTCC) 443 strain. Randomly dispersed AgNPs with different diameters traveled via diffusion and redistributed in the confined plate over time. Fick’s second law of diffusion can be used to develop an analytical solution in one-dimensional linear and radial space. For full and irreversible adsorption, Fick’s second law gives the time-dependent concentration profile as a function of the distance from the absorbing wall [9]:

(2) $$c\left(x,t\right)=c^{*}erf\left(\frac{x}{2\sqrt{Dt}}\right),$$

where c* is the bulk concentration. The concentration defined as the number of entities per volume can be interpreted as the probability of finding a particle in space. The underlying principle that allows such probability studies is that Brownian motion of particles in a solution, resulting from inter-particle collisions, is independent of diffusion. On the other hand, the concentration in a radial space is expressed by:

(3) $$c\left(r,t\right)=c^{*}\left[1-\frac{r_{s}}{r}erfc\left(\frac{r-r_{s}}{\sqrt{4Dt}}\right)\right],$$

where *r_s_* is the radius of the sphere. This relationship determines the probability of finding a particle in the distance *r* from the center of an absorbing sphere.

Compton group [10] applied a similar idea to calculate the probability of nanoparticle interactions with a sensor. They studied diffusional nanoimpacts by using one-dimensional random walk simulations in a very low concentration from 0.1 pM to 0.1 fM. The cumulative number of hits with the zone of one standard deviation is shown in a graph (Figure 1b). The estimated number of hits (${\hat{N}}_{hits}$) shows a strong prediction at low concentrations of particles where only a few hits are expected. In this prediction of analytical hits, many different types of sensors/electrodes can be considered. For example, typical electrode designs such as microwires and microdiscs were studied to provide a quantitative expectation of sensitivity via diffusional impacts of NPs [11]. The average number of hits (impacts) on a microwire electrode can be expressed by:

(4) $${\hat{N}}_{hits}\left(t\right)=2\pi p^{*}lr_{c}{}^{2}F^{*}\left(\tau \right),\mathrm{where}\;\tau =Dt/r_{c}{}^{2},$$

where p* is the NP concentration, *l* is the length of the wire, *r_c_* is the radius of the wire, and $F^{*}\left(\tau \right)$ is a time-dependent function. This equation was also used to calculate the first passage time of NPs on the electrode. This analytical study provided a quantitative basis to design a highly sensitive electrode for NP detection. In this study, they found that a microwire electrode has an advantage compared to a microdisc electrode. When the same surface area (6.28 nm^2^ in a concentration of 1 fM and a diffusion coefficient of 10^−11^ m^2^ s^−1^) was considered, a microwire electrode (radius of 1 μm) achieved a first passage time of 90 s, while the microdisc required 660 s. Collectively, diffusion-based detection of NPs depends on the diffusion coefficient (related to temperature and viscosity), electrode type and dimension, and sensing time. Thus, for a given sample with a specific diffusion coefficient, the sensing time determines the capability of a sensor. Consequently, a high NP hit probability requires extensive time, which is not ideal for time-sensitive molecular diagnostics both at laboratory and point-of-care settings.

The basic principle of diffusion has been used in viral particle detection. Typically, diffusion-based concentration methods utilize capture probes that bind with target viral particles at specific points in their natural motion. Most probes use either immobilized antibodies, which capture viral particles through antigen–antibody interactions or DNA hybridization probes, which consist of a specific single-stranded nucleotide sequence complimentary to the target viral ssDNA or RNA, or ligand-functionalized NP via Au plasmon shift [12]. Depending on the probe architecture, binding could result in viral particle aggregation [13,14,15,16,17,18,19], collection on a 2D or 3D structure [20,21,22,23,24,25,26], or simply the creation of an individually “labelled” viral particle [27,28]. The ultimate detection method depends on the unique experiment design. However, the two most common detection parameters are colorimetric intensity [14,15,18] and electrochemical interactions [20,21,22,26]. The biggest advantages of the diffusion-based methods are their relatively low sample volume and assay simplicity. Sample volume requirements are typically in the micro-liter scale, which is similar to that of ELISA, but requires fewer individual process steps [26]. 

In 2015, Zhang group demonstrated that influenza A virus (H3N2) infections could be detected rapidly without expensive analysis tools [15]. In their experiment, 13 nm gold nanoparticles (AuNPs) were incubated with anti-H3N2 monoclonal antibodies (mAb) at 37 °C for 2 h with gentle shaking. The antibodies adsorbed onto the AuNPs through ionic and hydrophobic interactions (Figure 1c). These mAb-AuNPs were then centrifuged, washed, and stored. Figure 1d shows that mAb-AuNPs induce aggregation in positive samples due to antigen–antibody binding. AuNPs exhibit surface plasmon resonance, thus aggregation resulted in a color shift from red to blue due to a larger mean particle diameter (Figure 1e). The detection limit for this method was determined to be 7.8 Hemagglutination units (HAU) in a 250 μL sample, with a process time of 35 min. This study showed a potential to work with several other antigen–antibody pairs such as HIV, hepatitis, or other influenza strains.

An optofluidic sensor (Figure 1f–h) from Altug group [26] uses a similar principle to immobilize antibodies onto a gold-plated nanohole (Figure 1f). This sensor detects small RNA viruses (vesicular stomatitis virus and pseudotyped Ebola) and large enveloped DNA viruses (vaccinia virus). This sensor was fabricated through a combination of electron-beam lithography, reactive ion etching, and metal deposition. The resulting sensor surface was then functionalized with protein A/G to facilitate the immobilization of three different antibodies: anti-VSV, anti-Ebola, and anti-vaccinia antibodies. When immersed in an infected sample, target viral particles adhere to the sensor through antigen-antibody binding (Figure 1g). Plasmon resonance determines the color of light that passes through this nanohole sensor, resulting in a resonance shift (Figure 1h). This group achieved an overall process time of 90 min with a high degree of specificity, but did not fully investigate the lower detection limit of this method.

Table 1 summarizes various viral detection methods using diffusion-based concentration. Weissleder group [17] demonstrated a very high detection sensitivity (1 viral particle/μL), but the process time was 120 min, which captures the intrinsic limitation of the passive nature of diffusion. In other words, viral particle concentration is only achieved through randomly catching target particles along their path, without any means of actively directing the target particles to the capture point. Mixing can be utilized to improve the overall diffusion rate [13,16], but ultimately this will influence process time more than detection limit. Collectively, active concentration methods are required to offer rapid and sensitive detection of viral particles.

### 2.2. Centrifugation and Microfiltration

**Figure 2:**
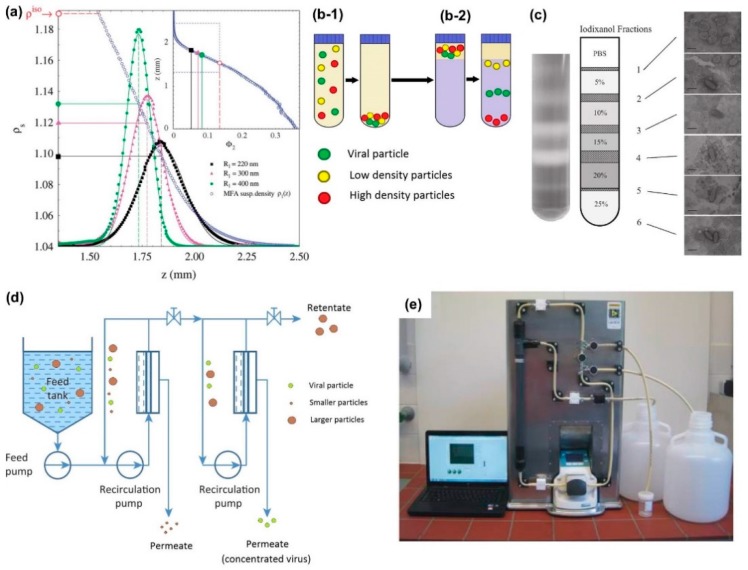
(**a**) Gaussian concentration profiles showing that concentration peak locations vary with particle density (reproduced from Piazza et al. [42]); (**b**) illustrations depicting a representative two-part centrifugation process: (**b-1**) a viral sample is centrifuged, forming a pellet of solid matter; (**b-2**) the pellet is then re-suspended over a cushion with specific density gradient and centrifuged again, separating particles by their density; (**c**) photo of a centrifuge tube (**left**) after purification of White Spot Syndrome virus from infected shrimp tissue and an illustration describing the Iodixanol density gradient. Lines projected from the illustration show corresponding TEM images (**right**) of each gradient fraction, yielding a unique distribution of subcellular debris at each fraction (reproduced from Dantas-Lima et al. [45]); (**d**) a representative filtration loop, in which the first membrane is used to remove particles smaller than the target viral particles, while the second membrane is used to remove particles larger than the target viral particles (modified from Jungbauer 2013); (**e**) photograph of a crossflow filtration loop used to concentrate MS2 bacteriophage and human adenovirus particles (reproduced from Pei et al. [48]).

Centrifugation is an active concentration process that uses centrifugal force to control or accelerate sedimentation [35]. High-density particles experience a much greater force than low-density particles, resulting in asymmetric particle migration. This concentration method is ideal for samples with a high number of total nanoparticles. In theory, the sedimentation phenomenon of nanoparticles via centrifugal concentration is in a non-equilibrium state due to the complex hydrodynamic process [36,37]. The dynamics of nanoparticles in a medium is chaotic, varied by initial conditions and diffusive behavior of particles induced by particle-interacted flows. Nevertheless, a modified Smoluchowski equation [38,39,40] describes the centrifugation-enabled sedimentation in non-equilibrium. The time-dependent concentration profile using non-dimensional parameters is described [41]:

(5) $$\partial_{t}c\left(z,t\right)=\partial_{z}\left(D\partial_{z}c(z,t\right)+\kappa c\left(z,t\right)F^{ext},$$

where *D* is the thermal diffusivity and *F^ext^* is the external force. As shown in Figure 2a, the concentration profile gives Gaussian distribution [42], such that the peak position of the Gaussian patterns follows to the position with the highest density of nanoparticles. As centrifugal concentration continues, the peak position shifts along with the overall distribution. Different shapes and dimensions of particles result in different Gaussian distributions, leading to separation. The average sedimentation velocity determines the shift rate of nanoparticles. The Svedberg coefficient (S) describes the sedimentation rate depending on the ratio of effective mass and friction factor:

(6) $$S=\frac{v}{\omega^{2}r}=\left(m-m_{o}\right)/\zeta ,$$

where *v* is the sedimentation velocity, *ω* is the angular speed, *r* is the particle distance, *m* is the particle mass and *ζ* is the friction coefficient. 

An illustration in Figure 2b explains the centrifugal concentration of viral nanoparticles with different densities. A single target in a high-density solution, known as a cushion [43,44], yields a concentrated pellet after centrifugation (Figure 2b-1). If specific particle separation is required, a combination of high-density solution layers can be used, also known as a gradient. This yields a fractionalized sample after centrifugation [45], shown as a schematic illustration in Figure 2b-2. In 2013, the Dantas-Lima group demonstrated that white spot syndrome virus (WSSV) can be purified and concentrated using a two-step centrifugation process [45]. First, they concentrated solid viral matter by centrifugation at 60,000× *g* for 2.5 h onto a 50% iodixanol cushion, which increased the viral particle concentration approximately 11 times. The resulting pellet (5 mL) was then re-suspended and centrifuged at 80,000× *g* for 3 h over an iodixanol gradient, comprised of several sections including phosphate-buffered saline (0% iodixanol), 5%, 10%, 15%, 20%, and 25% iodixanol. The result of the centrifugation-based particle concentration is summarized in Figure 2c. Each iodixanol fraction contains a combination of WSSV, extracellular debris, and other contaminants. By analyzing the contents of each fraction, researchers were able to characterize how the iodixanol gradient concentration affects the resulting sedimentation. They found that fraction 3 (10% iodixanol) demonstrated both the highest overall WSSV infectivity and the lowest concentration of protein (quantified via total protein assay) and cellular contaminants (quantified via TEM observation).

Microfiltration is another physical concentration process, in which a heterogeneous sample mixture is forced to pass across a membrane filter using a pump. Microfiltration membranes are typically categorized by their pore diameters, which will ultimately determine which particles pass (permeate) and which particles are withheld [46]. An overview of the microfiltration process of polydisperse particles in Figure 2d describes a representative loop using a series of membrane filter stages to separate target viral particles from a mixed suspension. Another widely used technical term, ultrafiltration, is essentially following the same physical process as the microfiltration, but it requires higher filtration pressure and membranes that are classified by their molecular weight cut-off rather than pore size [47]. Pei group [48] demonstrated that several representative viruses (MS2 bacteriophage, human adenovirus, and murine norovirus) could be successfully concentrated by using a two-step filtration method (experimental setup in Figure 2e). The first step involves processing of an infected water sample (10 L) through a hollow fiber ultrafiltration loop, with a pore size of 20 nm and a transmembrane pressure (TMP) of 0.2 bar. The total process time for the filtration is only 22 min, resulting in a volumetric concentration factor (VCF) of 100 with a recovery rate of 31 ± 8%. VCF is the ratio between original starting volume and final elution volume, while the recovery rate describes what percentage of the starting viral mass is present in the final elution volume [48]. In the second step, the sample is acidified with HCl to pH 3 before further processing through an epoxy-based monolithic affinity filter (MAF). This type of filter differs from previously described filters because it operates using chemical binding, rather than physical exclusion. With proper MAF composition, target viral particles are bound to the filter column, while non-target particles are passed. After capturing, the viral particles are eluted off the filter column with 1 mL of glycine-beef extract buffer (pH 9.5). The total process time for the second stage is only 11 min with an additional concentration factor of 100 and a recovery rate of 73.3 ± 6.3%, which makes the total process time of 33 min with an enhanced concentration factor of 10,000.

Table 2 summarizes recent research outcomes that used centrifugation and/or microfiltration-based methods to concentrate or purify a variety of viral particles. The major advantage of these methods is their ability to efficiently handle large sample volumes from the milliliter to liter scale. For example, these methods will be directly applicable in bulk processing of large water samples, such as aquatic forecasting and water quality characterization [45,48,49,50,52,54]. One common obstacle among bulk filtration techniques is filter clogging and fouling. Zhang group devised a lanthanum-based flocculation technique for reducing a 40 L MS2/adenovirus sample to 1 L prior to filtration, significantly reducing the impact of membrane contamination [54]. Wickramasinghe group demonstrated that human influenza A virus can be selectively concentrated using both microfiltration and ultrafiltration techniques [47]. They found that ultrafiltration through a 300 kDa membrane worked best, due to its ability to concentrate H1N1 particles in the retentate while also removing host cell proteins and DNA in the permeate. They also demonstrated that mixed viral samples could be fractionalized using a series of microfiltration steps with decreasing pore size.

Collectively, the aforementioned concentration methods are useful, but not ideal for simple, rapid diagnostic testing and point-of-care diagnostics due to the required large sample volume, equipment-heavy setup, and long process time. In addition, these methods heavily rely on conventional detection using plaque assay [49] or real-time quantitative polymerase chain reaction (RT-qPCR) [50,52]. Additionally, the centrifugation and filtration processes often subject analytes to a great degree of shear stress, which can damage target particles and increase contamination [46]. This effectively limits the lower sensitivity of these concentration steps, since they must account for some level of particle degradation.

### 2.3. Electric and Magnetic Fields

Recently, electric and magnetic fields have both been used to actively concentrate viral particles. Magnetic fields exert a force on ferromagnetic materials, such as iron, cobalt, and nickel [55]. The active concentration of target particles in a medium needs to consider all forces acting on the particles, which determines the particle velocity ($v_{p}$) based on the Newton’s law [56]:

(7) $$m_{p}\frac{\partial v_{p}}{\partial t}=F_{b}+F_{drag}+F_{m},$$

where $F_{b}$ is the buoyancy force (Equation (8)), $F_{drag}$ is the drag force (Equation (9)), and $F_{m}$ is the magnetic force (Equation (10)):

(8) $$F_{b}=V_{p}\left(\rho_{p}-\rho_{f}\right)g,$$

where $V_{p}$ is the volume of a single particle, $\rho_{p}$ and $\rho_{f}$ are the density of the particle and the medium, and g is the gravitational acceleration.

(9) $$F_{drag}=-3\pi \eta d_{p}v_{p},$$

where η is the fluid viscosity and $d_{p}$ is the core diameter of the particle.

(10) $$F_{m}=\left(m\cdot 𝛻\right)B=\frac{V_{p}\left(\chi_{p}-\chi_{f}\right)}{2\mu_{0}}𝛻B^{2},$$

where m is the magnetic dipole moment, B is the external magnetic field, 𝛻B is the gradient of magnetic field, $\chi_{p}$ and $\chi_{f}$ are the volume magnetic susceptibility of the particles and the fluid, respectively, and $\mu_{0}$ is magnetic permeability of air or vacuum. The combination of the listed Equations (7)–(10) provides a governing equation, describing the vertical velocity of a particle attracted by the magnetic field:

(11) $$v_{p}\left(z\right)=\frac{V_{p}}{3\pi \eta d_{p}}\left[\frac{\chi_{p}-\chi_{f}}{2\mu_{0}}𝛻B_{z}{}^{2}+\left(\rho_{p}-\rho_{f}\right)g\right].$$

**Figure 3:**
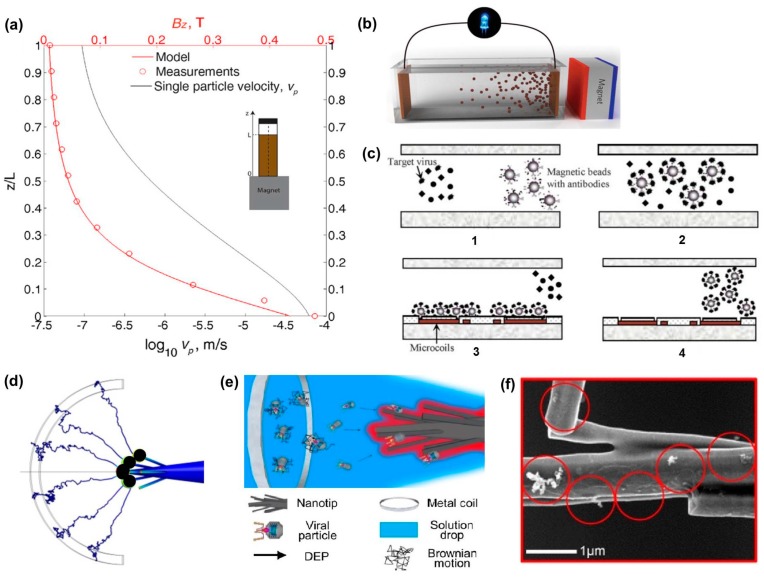
(**a**) graph showing an increase in particle settling velocity with an increase in magnetic field strength (reproduced from Prigiobbe et al. [56]); (**b**) illustration depicting the migration of Fe_3_O_4_ nanoparticles in solution under the influence of a magnetic field (reproduced from Yan et al., [57]); (**c**) illustration of a magnetically assisted concentration device for detection of Dengue virus (reproduced from Lien et al. [58]): (**c-1**) antibody-conjugated magnetic beads are introduced to the viral solution; (**c-2**) magnetic beads and viral particles bond; (**c-3**) bead-virus constructs are concentrated via electrically induced magnetic micro coils downstream; (**c-4**) constructs are released for analysis by shutting off the micro coils; (**d**) computational analysis showing particle trajectories, induced by dielectrophoresis (DEP), toward a dendritic nanotip; (**e**) illustration of a nanotip electrode for DEP concentration of viral particles; (**f**) representative SEM image of the nanotip with captured T7 phage particles (reproduced from Yeo et al. [63]).

**Figure 4:**
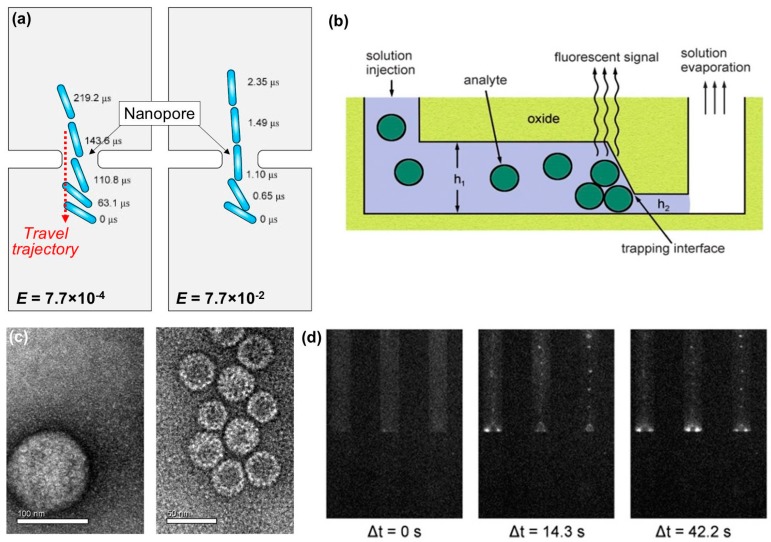
(**a**) diagram showing cylindrical particle trajectories through a nanopore, due to an electric field. A 100-fold increase in electric field strength (**right**) results in a 100-fold increase in speed (reproduced from Ai and Qian [91]); (**b**) illustration showing a general operation of a fluidic device, where h_1_ is the taller segment and h_2_ is the shorter segment that determines what size particles will be trapped; (**c**) images of Herpes simplex virus (HSV-1) capsids (**left**) with mean diameter of 125 nm and Hepatitis B virus (HBV) capsids (**right**) with mean diameter of 30 nm; (**d**) time-lapse fluorescence images of the working device showing an increase in fluorescent signal for trapped HSV-1 and HBV capsids (reproduced from Hamblin et al. [92]).

The measured $v_{p}$ of iron-oxide nanopaticles, along the axis of symmetry parallel to the z-axis, is shown in Figure 3a [56]. This graph clearly demonstrates that the particle settling velocity during concentration increases due to the strength of the gradient of magnetic field. Many research groups have found ways to functionalize ferromagnetic nanoparticles that capture target viruses or cells, allowing them to concentrate target particles using a magnetic field. A recent study [57] shows that a Fe_3_O_4_ nanoparticles in solution can be concentrated at a target electrode (Figure 3b) due to the induced magnetic field. Another example using a microfluidic chip [58] demonstrated the concentration of Dengue virus particles by using magnetic beads (Figure 3c). In this process, anti-dengue antibody-conjugated magnetic beads are mixed with a viral sample and incubated for 20 s (Figure 3c-1). During incubation, target viral particles bind to the magnetic beads, due to the antibody–antigen interaction, while undesired particles remain in solution (Figure 3c-2). Then, a direct current (DC) of 0.5 A is applied to the integrated microcoils, inducing a magnetic field that attracts the magnetic beads (Figure 3c-3). After collecting beads for 5 min, the channels are washed with phosphate buffered saline (Figure 3c-4). The overall concentration time for this device is only 10 min, while traditional bio-sample preparation for RT-PCR can take up to 48 min and carries a higher contamination risk [59]. The major improvement in the concentration time is due to the use of a magnetic field, which is the active element of this concentration technique.

Similarly, electric fields can also be utilized for effective and rapid concentration of target nanoparticles. Electric field-based concentration has versatility to control particle movement directions and speed in several ways, depending on the particle properties and field conditions. Electrophoresis (EP) describes the motion of dispersed charged particles relative to their suspension fluid under the influence of a uniform electric field [60]. The EP force (***F****_EP_*) is exerted on the charged particle, which is expressed as [61]:

(12) $$F_{EP}=q\times E,$$

where *q* is the charge of a particle and *E* is the electric field generated between two electrodes. The movement of charged particles in a medium to an electrode can be calculated in an electric field. The particle velocity (*v_EP_*) induced by the EP force is expressed as:

(13) $$v_{EP}=\frac{\varepsilon \xi_{p}E_{\infty }}{\eta },$$

where ε is the dielectric permittivity of a particle, $\xi_{p}$ is the zeta potential, $E_{\infty }$ is the uniform electric field, and η is the viscosity of a solution.

Similarly, dielectrophoresis (DEP) is used to actively control the motion of dispersed dielectric particles under the influence of a non-uniform electric field [62]. The trajectory of nanoparticles in a medium is studied by considering relevant forces including drag force, Brownian motion force, and DEP. The total force (***F****_N_*) is described by [63]:

(14) $$F_{N}=F_{Drag}+F_{Brownian}+F_{DEP}.$$

Here, the drag force (***F****_Drag_*) results from the relative motion of a spherical particle under a fluid flow in a solution:

(15) $$F_{Drag}=-6\pi \mu r\left(\frac{∆x}{∆t}-u\right),$$

where *μ* is the viscosity of medium, *r* is the particle radius, ***x*** is the particle displacement vector, *t* is the time, and ***u*** is the flow velocity. The Brownian motion-induced force (***F****_Brownian_*) is caused by random thermal fluctuation in medium [64]:

(16) $$F_{Brownian}=\sqrt{\frac{12\pi \mu rk_{B}T}{∆t}},$$

where *k_B_* is the Boltzmann constant, and *T* is the absolute temperature of a solution. The DEP force is calculated by the effective dipole moment theory [65]:

(17) $$F_{DEP}=2\pi r^{3}\varepsilon_{m}Re\left[CM\right]𝛻{\left|E\right|}^{2},$$

where *ε_m_* is the permittivity of medium, ***E*** is the electric field vector, and *CM* is the polarization Clausius–Mossotti factor. The CM factor is determined by the relative polarizability of a particle in fluid, which includes the frequency-dependent permittivities (ε_p_^*^ and ε_m_^*^) of a particle and medium, respectively. The CM factor for a sphere is:

(18) $$CM=\frac{\varepsilon_{p}{}^{*}-\varepsilon_{m}{}^{*}}{\varepsilon_{p}{}^{*}+2\varepsilon_{m}{}^{*}},\;\mathrm{where}\;\varepsilon^{*}=\varepsilon +\frac{\sigma }{i\omega },$$

where ε^*^, ε, *σ*, and *ω* are the complex permittivity, the DC permittivity, the conductivity, and the applied frequency, respectively. Collectively, the particle travel path and concentration speed can be estimated by substituting Equations (15)–(17) into Equation (14):

(19) $$∆x=u∆t+\frac{1}{6\pi \mu r}\left(F_{Brownian}+F_{DEP}\right)∆t.$$

Multiple particle paths in a medium can be investigated when they are concentrated to a sharp electrode by DEP (Figure 3d) where a nanostructured tip attracts nanoparticles, dispersed in a solution drop. This DEP method using an alternating current (AC) electric field has successfully concentrated low-abundance nanoparticles such as T7 phage [63,66], DNA [67,68], gold [69], and oligonucleotides [70,71]. A schematic illustration in Figure 3e [63] captures the working principle of DEP for concentration of nanoparticles in a medium. A dendritic tip, comprised of silicon carbide nanowires wrapped with single-walled carbon nanotubes, is immersed in a 2 μL sample droplet opposite a metal coil. When an AC potential (20 peak-to-peak voltages at 5 MHz) is applied between the tip and metal coil, each dendritic branch generates its own electric field, resulting in a strong non-uniform electric field overall. The resulting DEP force is large enough to overcome the Brownian motion of viral particles (Figure 3d), resulting in viral buildup on the tip (Figure 3f). The process time for this method is only 5 min (limited by droplet evaporation) with a lower detection limit of 10^4^ particles/mL.

Table 3 summarizes several additional groups that have used magnetic and electric field-based methods to concentrate viral particles. Some groups modified conventional capillary gel electrophoresis techniques to detect specific genomic segments, including those of influenza virus, West Nile virus, SARS coronavirus, Dengue virus, and more [72,77,78,80]. Other groups focused on the concentration and detection of viral particles by using either DEP methods or functionalized magnetic NPs [58,63,73,74,76,79]. A research group was even able to demonstrate single viral particle detection [75] with rapid process time. Overall the greatest advantage of these methods is the rapid process time, all of which were below 30 min. While most groups did not demonstrate a detection limit significantly lower than that of diffusion-based methods, Iwata group developed a method of concentrating viral RNA with sulfonated magnetic beads to reduce the RT-PCR detection limit as low as 10^2^ viral copies/mL [73]. It is worth noting that many biological analytes can be directly manipulated with an electric field, resulting in EP, DEP, or a combination of both. Furthermore, the electrode geometry and electric field properties can be tuned to maximize their performance with respect to a given analyte [83]. On the other hand, very few biological analytes are naturally ferromagnetic, requiring an additional functionalization step [84]. All else being equal, this implies that electric field-based methods will ultimately prove to be faster and more versatile than magnetic field-based methods.

### 2.4. Nano-Microfluidics and Other Emerging Technologies

Nano-microfluidic devices incorporate a variety of functions such as mixing, sample incubation, particle concentration and detection all in one device [85,86]. This is accomplished by utilizing engineered nano-microscale channels, pores, pumps, and valves in conjunction with electromagnetic or electrochemical sensors. At the same time, these miniaturized, low-profile devices require minimal sample volumes for analysis, which provides several advantages including low energy consumption, rapid heating and cooling cycles, precise sample control, and relatively fast assay time [2]. For these reasons, sufficiently complex microfluidic devices are often referred to as LOC devices [87]. Many fluidic devices utilize one or several of the concentration methods including diffusion, microfiltration, electric fields, and magnetic fields. A few examples show molecular diffusion by pressure-driven laminar flow [88], diffusion-based colorimetric assays [89], magnetic bead conjugation and concentration [4], and precise electromagnetic particle detection [90]. The integration and automation of these methods and processing steps enables portable, point-of-care LOC devices.

Nano-microfluidic devices use fluid flow to deliver target nanoparticles in a medium via channels or pores. The Reynolds number of flow in nano-microscale structures is very small, which gives negligible inertial terms in Navier–Stokes equations [91]:

(20) 𝛻·u=0,

(21) $$Re\frac{\partial u}{\partial t}-{𝛻}^{2}u+𝛻p-2sinh\psi 𝛻\left(\psi +\phi \right)=0,$$

where u is the fluid velocity vector, Re=ρUa/μ is the Reynolds number (ρ is the fluid density, U is the fluid velocity, a is a characteristic dimension, and μ is the fluid viscosity), p is the pressure, and the last term describes the electrostatic force from the interaction between the overall electric field and net charge within the electrical double layer. Here, Newton’s second law describes the translational velocity of nanoparticles that are concentrated via nanopores (Figure 4a):

(22) $$m\frac{dU}{dt}=F_{E}+F_{H},$$

(23) $$F_{E}=2{\left(\kappa a\right)}^{-2}\int^{\text{​}}T^{E}\cdot nd\Gamma ,$$

(24) $$F_{H}=\int^{\text{​}}T^{H}\cdot nd\Gamma ,$$

where *m* is the particle’s mass, $F_{E}$ is the electrical force, $F_{H}$ is the hydrodynamic force, 1/κ is the Debye length, $T^{E}$ is the Maxwell stress tensor, $T^{H}$ is the hydrodynamic stress tensor, and Γ is the dimensionless particle surface. Figure 4a shows particles in a fluid passing through a nanopore with different travel speeds, caused by electric field (E). When the electric field is increased 100 times (right, Figure 4a), the translocation velocity is increased about 100 times, compared to the lower electric field case (left, Figure 4a). The aforementioned equations provide the basic principles to describe fluid flow characteristics and particle trajectory in nano-microfluidics. 

In 2010, selective concentration of viral particles (Herpes simplex virus 1 (HSV-1) and Hepatitis B virus (HBV)) was demonstrated in dual-height nanofluidic channels via physical trapping [92]. Figure 4b illustrates the working principle of this device with target analytes in a injected solution. Fluidic flow is induced in a 20 μm-wide channel via capillary action. Once the channel is filled, then flow is limited by evaporation, while particles that are too large to pass through the shallow outlet (with height h_2_ in Figure 4b) become trapped. This method is similar to that of microfiltration, in that each trapping interface acts like a membrane pore which limits the movement of particles too large to pass. However, this method also presents several advantages over a typical microfiltration loop. It can handle much smaller sample volumes (as low as 200 μL) and does not require any applied external pressure. Furthermore, the detection step takes place in real time provided the target nanoparticles can be visualized. In this case, HSV-1 and HBV capsids (Figure 4c) were fluorescently labeled and their aggregation at the trapping interfaces was observed over the course of 42.2 s, as shown in Figure 4d.

Table 4 summarizes recent reports that have investigated nano-microfluidic devices for viral particle detection. Each of these groups demonstrated process times of less than one hour, with minimum sample volume requirements of less than 400 μL. Numerous groups were able to achieve detection limits lower than or equivalent to that of comparable commercial viral assays, such as ELISA, flow cytometry, and hemagglutination assay, while minimizing the risk of contamination by automating process flow. In general, most of these devices fall into one of two categories. The first category consists of devices that use passive microstructures to mechanically limit particle movement. This is a relatively new approach and is far less common than the second category, which consists of devices that use complex process automation in order to reduce sample requirements and improve overall assay time and sensitivity. One such example is the compact-disk device, which facilitates whole blood injection, sample preparation, reagent mixing, and particle detection in one device [98]. This group was able to detect HBV from whole blood in less than thirty minutes, with a detection limit 6.5 times lower than that of real-time qPCR [34]. This work highlights the greatest advantage of LOC devices—the automated integration of both passive and active process steps into a single device. Thus, microfluidic devices are suitable for integrating sample preparation and processing into a single device, while also allowing us to investigate novel means of physical nanoparticle manipulation using fluid mechanics and trapping principles.

**Figure 5:**
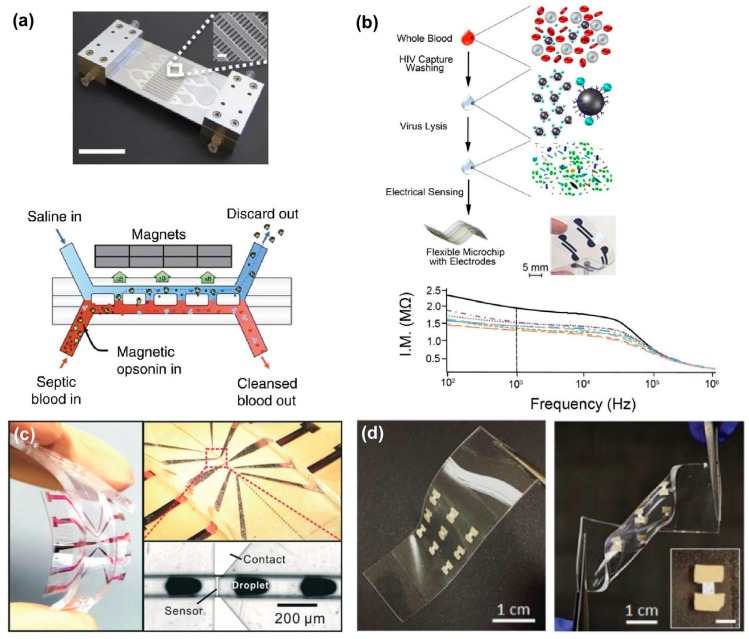
(**a**) a microfluidic device (**top**) for removing pathogens from blood in real-time with illustration (**bottom**) describing working principles, which combine both passive and active concentration methods to capture and remove target particles (reproduced from Kang et al., [102]); (**b**) illustration describing a flexible sensor for HIV detection. Lysed viral contents change the solution conductivity, which is measured with integrated electrodes and used to characterize the level of infection (reproduced from Shafiee et al. [103]); (**c**) photographs of a flexible giant magnetoresistive (GMR) analytical device which senses magnetic nanoparticle emulsion droplets on-chip (reproduced from Lin et al. [104]); (**d**) photographs of a flexible photodetector array demonstrating bending and twisting deformation modes (reproduced from Sharma and Ahn [105]).

Due to the advantages present in fluidic assemblies, the next generation of viral diagnostic tools will most likely take the form of various LOC devices. Figure 5 highlights several capabilities demonstrated by modern nano-microfluidic devices. The device illustrated in Figure 5a utilizes functionalized magnetic nanoparticles to remove a broad range of pathogens from whole blood in a manner similar to the magnetic concentration methods [102]. This technique could reasonably be modified to target specific viral particles, enabling real-time concentration, detection, and even removal through a single platform. In addition to automating concentration and detection techniques, there has been a recent focus on developing flexible LOC devices (Figure 5b–d).

Flexibility is one of the key characteristics for enabling integrated non-invasive devices that can bend and stretch to accommodate the dynamic contours of a human body [1]. The flexible device shown in Figure 5b utilizes functionalized nanoparticles to capture HIV particles on the chip. After washing and viral lysis, the integrated electrodes are used to detect a change in solution conductivity, indicating the presence of HIV [103]. The flexible analytical device shown in Figure 5c uses a giant magnetoresistive multilayer to count emulsion droplets carrying magnetic nanoparticles, and demonstrated full performance with multi-modal bending [104]. Similarly, the photodetector array shown in Figure 5d is also capable of maintained performance despite extreme bending and stretching [105]. These examples account for electrical, magnetic, and light-sensitive flexible sensors, each of which may soon find applications in integrated LOC devices for viral diagnostics.

It should be noted that this review has not thoroughly covered the specificity of the discussed methodologies due to the lack of relevant data in most of the references. Many of those articles mention about a high specificity of viral detection qualitatively; for instance, functionalized antibodies and hybridization probes interact specifically with target proteins and genetic sequences in a lock-and-key manner. 

## 3. Conclusions and Future Perspectives

Recent study in nanomaterials and nano-microstructures has resulted in novel methods of concentrating and manipulating nanoparticles. In this review, we summarized the primary mechanisms in viral particle concentration and detection by utilizing diffusion, centrifugation and microfiltration, electric and magnetic fields, or nano-microfluidics. Diffusion-based methods offer a simple and cost-effective solution for particle concentration due to their passive nature, yet require extensive processing time. Centrifugation and microfiltration techniques provide unique advantages when handling large volume samples, but necessitate expensive and complex systems in their execution. On the other hand, active particle manipulation with electric and magnetic fields has shown rapid and sensitive detection capabilities. Nano-microfluidic systems bring a portable, LOC environment to the detection of viral particles, which is enabled by a miniaturized, integrated platform for concentration methods and automating multiple process steps. In the resource-limited settings, such sensitive, portable, and simple devices are urgently required for clinical management. Soft materials and advanced 2D nano-microstructures enable the development of low-profile implantable or wearable biosensors and bioelectronics for point-of-care or long-term disease diagnostics and health monitoring. When integrated with wireless energy harvesting and telemetry systems, such in vivo biosystems would serve as a stand-alone LOC platform. Collectively, we believe that the union of novel nanoparticle concentration methods and miniaturized, flexible LOC devices will open up a new era of real-time detection of targets to minimize the transmission and severity of viral infections. 

## Figures and Tables

**Table 1:** Viral detection methods via diffusion, grouped by their reported detection unit ^(1)^.

Detection Unit	Target(s) [Ref]	Process Time	Sample Size	Limit of Detection (LOD)	Commercial LOD
	HBsAg [20]	95 min	10 μL	10^4^ fg/μL	0.7 fg/μL ^(2)^
[mass]	H1N1, H5N1, H7N9 [24]	120 min	n/a	1 fg/μL	
	H1N1 [25]	30 s ^(3)^	0.1 mL	2 × 10^−3^ fg/μL	
	RSV-A2, RSV-dG [27]	30–60 min	n/a	1 vp ^(4)^	10^2^ vp/μL ^(5)^
[viral particles (vp)]	HSV-1 [21]	45 min	1 μL	10 vp/μL	
	HSV-1, ADV-5 [17]	120 min	100 μL	1 vp/μL ^(6)^	
	HCV RNA [18]	30 min	7 μL	7.14 vp/μL	
[plaque forming units (pfu)]	F-RNA coliphages: MS2, QB, GA, HB-P22 [16]	180 min	140 μL	10^−3^ pfu/μL (MS2, QB) 10^−4^ pfu/μL (GA, HB-P22)	10 pfu ^(7)^
	VSV-pseudotyped Ebola, Vaccinia virus [26]	90 min	n/a	10^4^ pfu/μL ^(8)^	
[Hemagglutination Units (HAU)]	H3N2 [15]	35 min	200 μL	0.04 HAU/μL	0.1 HAU/μL ^(9)^
	H3N1 [19]	n/a	60 μL	2 × 10^−4^ HAU/μL	
[50% Tissue Culture Infective Dose (TCID)]	H1N1, H3N2 [13]	40 min	90 μL	10^2^ TCID_50_/mL	200 TCID_50_/mL ^(10)^
[International Units (IU)]	α-HBsAg IgG antibodies [22]	5 min	25 μL	3 × 10^−3^ IU/mL	56 IU/mL ^(11)^
n/a	Influenza B/Victoria [14]	10 min	n/a	0.09 vol %	n/a

^(1)^ Results based on pure or spiked serum samples; ^(2)^ Experimental detection limit for ELISA [29,30]; ^(3)^ For concentrations above 10^10^ particles/mL. Lower concentrations may take longer; ^(4)^ Theoretical lower limit, but not demonstrated; ^(5)^ Experimental detection limit for flow cytometry [31]; ^(6)^ Only for HSV-1; ADV-5 lower sensitivity limit was not investigated; ^(7)^ Experimental detection limit for plaque assay [32,33]; ^(8)^ Lowest demonstrated limit; potential lower limit <10^2^ pfu/μL; ^(9)^ Experimental detection limit for hemagglutination assay [33]; ^(10)^ Experimental detection limit for endpoint dilution assay [32]; ^(11)^ Experimental detection limit for HBV qPCR [34]. (-sAg means surface antigen, RNA means ribonucleic acid, IgG means immunoglobulin G).

**Table 2:** Viral concentration methods via centrifugation and/or microfiltration.

Target(s) [Ref]	Sample Size	Sample Type	Volumetric Concentration Factor
somatic coliphage [49]	400 mL	seawater	80
white spot syndrome virus [45]	60 mL	shrimp tissue	12
Ostreid herpesvirus-1 [50]	15 mL	seawater	15
Semliki Forest virus [43]	29 mL	pure	4.1
VSV-G [51]	30 mL	pure	5 ^(1)^
Salmon amaemia virus [52]	0.2 mL	pure	n/a
HIV gag baculovirus [44]	2 L	pure	6
P22 Bacteriophage [53]	1 L	waste water	4
MS2, human adenovirus, murine norovirus [48]	10 L	pure	10^4^
H1N1 [47]	585 mL ^(2)^	pure	5.3
MS2, mouse adenovirus [54]	40 L	spiked tap water	1.3 × 10^4^

^(1)^ Four centrifugation cycles of 90 min each. Additional cycles may concentrate further, since plateau was never reached; ^(2)^ Average volume across five trials. Actual volume ranged from 540 to 634 mL.

**Table 3:** Viral detection methods via magnetic and/or electric field, grouped by detection unit. ^(1)^

Detection Unit	Target(s) [Ref]	Process Time	Sample Size	Limit of Detection (LOD)	Commercial LOD
[mass]	hemagglutinin (HA1, HA2) [72]	22 min	20 μL	7 ng/μL ^(2),(3)^	0.7 fg/μL
[viral particles]	porcine parvovirus (PPV), poliovirus [73]	n/a	1–10 mL	10^2^ vp/mL	10^5^ vp/mL
	SV-40, HSV-1, PPV, poliovirus, HAV, HBV, HCV [74]	15 min	1 mL	10^3^ vp/mL^(2)^	
	T7 bacteriophage [63]	5 min. ^(4)^	2 μL	10^4^ vp/mL	
	Influenza A [75]	10–20 min	n/a	1 vp	
	Influenza virus [76]	5 min ^(5)^	n/a	1 vp	
[plaque forming units]	Dengue virus (DENV) [58]	10.5 min	25 μL	10^−1^ pfu/μL	10 pfu
[genomic copies]	West Nile virus, Saint Louis encephalitis virus, JEV, Western/Eastern equine encephalomyelitis viruses [77]	n/a	5 μL	10^2^ RNA copies/μL ^(2)^	8–12 genomic copies ^(6)^
	SARS, DENV, JEV, Influenza A, human adenovirus [78]	20 min	1 μL	6 × 10^2^ DNA copies/μL ^(7)^	
[moles]	Japanese encephalitis virus (JEV) [79]	n/a	2 mL	0.32 nM	n/a
n/a	Influenza A (H7N7) [80]	320 s	1 μL	n/a	n/a

^(1)^ Results based on pure or spiked serum samples; ^(2)^ Lowest demonstrated. Lower limits were not fully characterized; ^(3)^ Note that hemagglutinin mass is not directly comparable to viral mass; ^(4)^ Process time limited by evaporation of 2 μL sample; ^(5)^ Process time to infect a single cell, visually confirmed under microscope; ^(6)^ Experimental detection limit for RT-qPCR [81,82]; ^(7)^ Lower detection limit of a capillary gel electrophoresis DNA sequencer.

**Table 4:** Viral detection methods via nano-microfluidics, grouped by detection unit.

Detection Unit	Target(s)	Process Time	Sample Size	Sample Type	Limit of Detection (LOD)	Commercial LOD
[mass]	HBV [90]	60 min.	n/a	plasma, serum	0.2 fg/μL ^(1)^	0.7 fg/μL
	Dengue Virus [89]	20 min	400 μL	saliva	20 fg/μL	
[viral particles]	Norovirus [93]	n/a	100 μL	pure	10^4^ vp/μL	10^2^ vp/μL
	HSV-1, HBV [92]	42 s ^(2)^	200 μL	pure	10^7^ vp/μL ^(3)^	
	HBV [94]	n/a	50 μL	serum	1 vp/μL	
[genomic copies]	HBV, E. coli [95]	12 min	100 μL	whole blood	10 DNA copies/μL	8–12 genomic copies
[Hemagglutination Units]	Influenza A [4]	15 min	25 μL	whole blood, serum, saliva	2 × 10^−5^ HAU/μL	0.1 HAU/μL
	H1N1 [96]	30 min	10 μL	pure	3.2 × 10^−3^ HAU/μL	
[50% Tissue Culture Infective Dose]	Bovine viral diarrhea virus (BVDB) [97]	<5 min	20 μL	pure	10 TCID_50_/mL	200 TCID_50_/mL
[International Units]	HBV [98]	<30 min	150 μL	whole blood	8.6 × 10^−3^ IU/mL	56 IU/mL
[moles]	Dengue Virus DNA (serotypes I, II, III, IV) [99]	90 s	1 μL	pure	100 pM	n/a
n/a	HIV-1 [100]	40 min	1 mL ^(4)^	plasma	n/a	n/a
	Dengue Virus RNA [101]	15 min	n/a	pure	n/a	n/a

^(1)^ Approximately equivalent to .05 IU/mL according to cited calculations; ^(2)^ Assay time only, does not including test sample preparation; ^(3)^ Approximate detection limit suggested by signal-to-noise ratio analysis, not demonstrated; ^(4)^ Largest sample size demonstrated. Smaller samples can be processed in proportionately less time.
